# Correlation between the female pelvic floor biomechanical parameters and the severity of stress urinary incontinence

**DOI:** 10.1186/s12894-023-01375-7

**Published:** 2023-11-30

**Authors:** Erzsébet Koroknai, Dávid Rátonyi, Krisztina Pákozdy, Attila G. Sipos, Zoárd Krasznai, Peter Takacs, Bence Kozma

**Affiliations:** 1 Department of Obstetrics and Gynecology, Faculty of Medicine, University of Debrecen, Nagyerdei krt. 98, Debrecen, 4032 Hungary; 2 Department of Obstetrics and Gynecology, Division of Female Pelvic Medicine and Reconstructive Surgery, Eastern Virginia Medical School, 825 Fairfax Avenue, Suite 526, Norfolk, VA 23507-2007 USA

**Keywords:** Stress urinary incontinence, Pelvic floor, Biomechanical parameters of the pelvic floor, Questionnaires, Disease severity

## Abstract

**Background:**

Stress urinary incontinence (SUI) is a common condition that requires proper evaluation to select a personalized therapy. Vaginal Tactile Imaging (VTI) is a novel method to assess the biomechanical parameters of the pelvic floor.

**Methods:**

Women with SUI were enrolled in this cross-sectional study. Participants completed the Medical, Epidemiologic, and Social Aspects of Aging (MESA) questionnaire and the Patient Global Impression of Severity Question (PGI-S) and underwent a VTI examination. Based on the MESA and PGI-S questionnaires, participants were divided into mild, moderate, and severe SUI groups. Fifty-two biomechanical parameters of the pelvic floor were measured by VTI and compared between the groups (mild vs. moderate and severe). SUI Score and Index were calculated from the MESA questionnaire. Pearson correlation was used to determine the strength of association between selected VTI parameters and the MESA SUI Index and MESA SUI Score.

**Results:**

Thirty-one women were enrolled into the study. Significant differences were observed in the VTI parameters 16, 22–24, 38, 39 when the difference between mild and severe subgroups of SUI based on the PGI-S score was examined. Parameter 16 refers to the maximum gradient at the perineal body, parameter 22–24 refers to the pressure response of the tissues behind the vaginal walls, and parameter 38, 39 refers the maximum pressure change and value on the right side at voluntary muscle contraction. VTI parameter 49, describing the displacement of the maximum pressure peak in the anterior compartment, showed a significant difference between the mild SUI and the moderate-severe SUI according to the MESA SUI score (mean ± SD 14.06 ± 5.16 vs. 7.54 ± 7.46, *P* = 0.04). The MESA SUI Index and SUI Score displayed a positive correlation concerning VTI parameters 4 (the maximum value of the posterior gradient) and 27 (the displacement of the maximum pressure peak in the anterior compartment) (VTI4 vs. MESA SUI Index r = 0.373, *P* = 0.039; VTI4 vs. MESA SUI Score r = 0.376, *P* = 0.037; VTI27 vs. MESA SUI Index r = 0.366, *P* = 0.043; VTI27 vs. MESA SUI Score r = 0.363, *P* = 0.044).

**Conclusions:**

Female pelvic floor biomechanical parameters, as measured by VTI, correlate significantly with the severity of SUI and may help guide therapeutic decisions.

**Supplementary Information:**

The online version contains supplementary material available at 10.1186/s12894-023-01375-7.

## Background

Urinary incontinence (UI) is a common disorder characterized by involuntary urine leakage, with a prevalence of up to 44–57% in middle-aged postmenopausal women and an increasing trend worldwide [1, 2]. UI causes physical, emotional, and social distress and severely restricts lifestyle and work activities [3]. Moreover, UI not only has an impact on the individual but also has a significant economic burden, with an estimated direct cost of 19.5 billion USD in the USA [4]. The two main UI types are stress urinary incontinence (SUI) and urge urinary incontinence (UUI). While SUI is a complaint of involuntary loss of urine on effort or physical exertion, including sporting activities, or on sneezing or coughing, UUI is a complaint of involuntary loss of urine associated with urgency [5]. The most common type is SUI, affecting approximately 46% of women, according to a nationally representative survey of US women [6]. However, most experts agree that the two main mechanisms of SUI are intrinsic sphincter deficiency (ISD) and urethral hypermobility [7]. These theories have remained broadly unchanged over the past decades, but they cannot accommodate individual variation and personalized medicine, and therefore, the precise elucidation of the pathophysiology, which varies slightly from individual to individual, is often not possible. Without an accurate understanding of the pathophysiology, the effectiveness of a therapy that is otherwise correctly administered according to a protocol may be reduced. For proper diagnosis and evaluation, a thorough history is essential. However, obtaining accurate data is often difficult, as patients are reluctant to talk about their urinary complaints or cannot provide precise information about the exact course of the disease, though this is crucial information, as it can determine the treatment choice. For that reason, the different questionnaires are helpful for an accurate assessment of complaints and disease severity. Two commonly used questionnaires in the investigation of incontinence are the subtype of the Medical, Epidemiologic, and Social Aspects of Aging (MESA) and the Patient Global Impression of Severity Question (PGI-S). MESA questionnaire has been created and validated to determine the predominant component (either urgency or stress) of mixed urinary incontinence (MUI) and evaluate the intensity of symptoms [8]. The PGI-S question is a validated universal measure used to evaluate the severity of a specific condition through a single-state scale [9]. These validated questionnaires are the standard tools for assessing UI, which can be used to assess the effectiveness of newer diagnostic tools and measures.

Vaginal Tactile Imaging (VTI) is a novel technology that creates a visual map of the female pelvic floor based on its biomechanical properties [10]. The visualization is based on 52 parameters, from which the instrument generates the Biomechanical Integrity (BI) Score, which in itself characterizes the biomechanical status of the female pelvic floor [11]. This approach, namely the BI-score, can become a diagnostic parameter in certain diseases has already been validated in pelvic organ prolapse (POP) [12]. Several international professional societies have formulated care protocols for stress incontinence. One common feature is that treatment of mild incontinence is preferable to non-medical care, such as lifestyle modification or weight reduction [13]. Like these, pelvic floor muscle training (PFMT) is particularly important for less severe incontinence but is no longer a sufficient therapeutic step for more severe incontinence. Conversely, if the patient has more severe incontinence symptoms, care requiring medical intervention is often considered necessary in addition to the former. Until now, there has been no known method for assessing the severity of incontinence other than questionnaires and pad-test that are simple, rapid, cost-effective, and non-invasive. Urodynamic investigation (UDI), as the gold-standard method, involves an economic burden, and its availability is limited in many places [14]. Therefore our study aimed to investigate the correlation between the female pelvic floor biomechanical parameters and the severity of stress urinary incontinence. We hypothesized that some biomechanical parameters of the female pelvic floor measured by the VTI correlate with the disease’s severity as measured by the MESA questionnaire and PGI-S.

## Methods

We conducted a cross-sectional study at an outpatient clinic between 4/1/22 − 15/3/22 at the University of Debrecen, Department of Obstetrics and Gynecology authorized under approval from the Scientific and Research Ethics Committee of Hungary: 2876-13/2022/EÜIG. After taking a medical history, potentially eligible patients completed the MESA questionnaire [8]. Women with stress or stress-predominant mixed UI (stress percent score more than urge percent score) on the questionnaire were selected for the study. Exclusion criteria were pregnancy or less than 12 months postpartum; more than three vaginal deliveries or prior operative delivery; pelvic organ prolapse (POP) > stage 2 prolapse according to the pelvic organ prolapse quantification system (POP-Q); and current medications for UI or prior surgical treatment for UI; collagen or connective tissue disease. POP was assessed by a standardized POP-Q examination after the International Continence Society recommendations [15]. After enrollment based on the MESA questionnaire, women were asked to complete the Patient Global Impression of Severity question (PGI-S). After a standardized pelvic exam, the biomechanical integrity of the pelvic floor was evaluated by VTI [11]. A vaginal examination was performed in all cases as part of the standardized pelvic exam, during which the POP-Q was recorded as described above.

### Questionnaires

#### Medical, epidemiologic, and social aspects of aging questionnaire

The questionnaire is developed and validated to identify the urgency- or stress-predominant component of MUI and assess the severity of symptoms [8]. The MESA is a self-reported questionnaire with nine questions on stress incontinence and six on urge incontinence. The four response categories range from “never” (0 points) to “often” (3 points), with higher scores indicating more frequent symptoms of incontinence. We calculated the stress score (maximum = 27 points), the urge score (maximum = 18 points), the stress index, and the urge index, which are obtained by dividing each category’s actual score by the maximum total possible. The MUI is categorized as stress-predominant if the stress index exceeds the urge index. To assess the severity of incontinence, the total score of stress (27) are divided into third degrees: scores 1 to 9 are assigned as mild, 10 to 18 moderate, and 19 to 27 as severe.

#### Patient global impression of severity question

The PGI-S is a universal measure utilized to assess the severity of a particular condition using a single-state scale. It has been validated specifically for women experiencing stress urinary incontinence [9]. The PGI-S scale ranges from 0, indicating the absence of symptoms, to 4, indicating the presence of severe symptoms, with a minimal clinically important difference (MID) of 1 [16].

### Vaginal tactile imager [11]

The biomechanical mapping of the pelvic floor was conducted using the Vaginal Tactile Imager (VTI), model 2 S (Advanced Tactile Imaging, Inc., Ewing, NJ, USA). This device is equipped with 96 consecutive pressure (tactile) sensors positioned on both sides of the probe, along with an orientation sensor and temperature controllers that maintain the probe’s temperature close to that of the human body during the examination. Throughout the clinical procedure, the VTI probe captures pressure responses from the opposing vaginal walls along the length of the vagina. By integrating the acquired pressure and positioning data from each pressure-sensing element, the tactile images of the vagina provide a comprehensive depiction of vaginal wall deformation and pelvic muscle contraction. The VTI examination procedure comprises eight tests, outlined as follows: (1) probe insertion, (2) elevation, (3) rotation, (4) Valsalva maneuver, (5) voluntary muscle contraction (anterior vs. posterior compartments), (6) voluntary muscle contraction (left vs. right side), (7) involuntary relaxation, and (8) reflex muscle contraction (cough). Tests 1, 2, 4, 5, 7, and 8 provide data for the anterior/posterior compartments, Test 3 provides data for a 360-degree evaluation, and Test 6 provides data for the left/right sides. The VTI probe allows for 3–15 mm tissue deformation during probe insertion (Test 1), 20–45 mm tissue deformation during probe elevation (Test 2), and 5–7 mm deformation during probe rotation (Test 3). Additionally, it records dynamic responses during the Valsalva maneuver, pelvic muscle contractions, and relaxation (Tests 4–8). The probe maneuvers performed during Tests 1–3 accumulate multiple pressure patterns from the tissue surface, enabling the creation of an integrated tactile image for the investigated area using image composition algorithms. Within the acquired tactile images of Tests 1 and 2, the software calculates spatial gradients (∂P(x, y)/∂y) for the anterior and posterior compartments. The y-coordinate represents the orthogonal direction from the vaginal channel, spanning the anterior-posterior compartments, while the x-coordinate is located on the vaginal channel itself. The VTI software automatically calculates 52 parameters across the eight test procedures listed separately in the appendix. We followed the manufacturer’s instructions in all study aspects as in previous published studies [17, 18].

### Statistics

For statistical calculations, we used IBM SPSS Statistics for Windows Version 25.0 statistical software (IBM Corp., Armonk, NY, USA). Descriptive statistics were computed for all variables of interest. The continuous outcomes were summarized using means and standard deviations, while categorical outcomes were presented as frequencies and percentages. Student’s t-test was used to compare mean values between two groups. Simple correlation analysis was used to examine the bivariate associations between selected parameters, indexes, and scores, and Pearson Correlation Coefficient (r) was calculated. Statistical significance was defined as a two-tailed test with a *P*-value threshold of < 0.05.

## Results

Thirty-one women participated in our study, and Table 1 provides an overview of their demographic and clinical characteristics. The mean body mass index (BMI) was 27.6 ± 5.8 kg/m2. Among the participants, 18 patients (58%) were postmenopausal. In terms of incontinence severity assessment, the MESA questionnaire and PGI-S questions were utilized. According to the MESA questionnaire, seven patients (23%) had mild SUI, 15 patients (48%) had moderate, and nine patients (29%) had severe. Although all patients reported symptoms of stress incontinence as their main complaint, if the total score of the urge domain of the MESA questionnaire exceeded two, those patients were classified as mixed incontinence (MUI n = 13, 42%). As per the PGI-S question, eight patients (26%) experienced mild incontinence, while 23 (74%) reported severe incontinence. Upon conducting a bivariate analysis to evaluate the associations between 52 VTI parameters and the MESA SUI Index, MESA SUI Score, MESA UUI Score, MESA UUI Index, and PGI-S Score, only the following correlations were found to be statistically significant (Table 2). VTI parameters 4 and 27 displayed a moderate positive correlation concerning the MESA SUI Index and MESA SUI Score (VTI parameter 4 vs. MESA SUI Index r = 0.373, *P* = 0.039; VTI parameter 4 vs. MESA SUI Score r = 0.376, *P* = 0.037; VTI parameter 27 vs. MESA SUI Index r = 0.366, *P* = 0.043; VTI parameter 27 vs. MESA SUI Score r = 0.363, *P* = 0.044). Specifically, parameter 4 represents the maximum value of the posterior gradient, indicating the pressure change per posterior wall displacement in a direction orthogonal to the vaginal channel. Parameter 27 refers to the displacement of the maximum pressure peak in the anterior compartment. Significant differences were observed when examining the distinction between mild and severe subgroups of SUI based on the PGI-S score, as shown in Table 3. Specifically, VTI parameters 16, 22, 23, 24, 38, and 39 demonstrated statistically significant differences between these two subgroups. Describing the above, parameter 16 refers to the maximum gradient at the perineal body posteriorly and can be interpreted as the strength of Level III support; parameter 22, 23, and 24 refers to the pressure response of the tissues and muscles behind the left and right vaginal wall; parameter 38 and 39 refers the maximum pressure change and value on the right side at voluntary muscle contraction which can be interpreted as a contraction strength of specific pelvic muscles. Patients with stress incontinence were categorized according to the severity of incontinence as mild and moderate or severe according to the MESA score, as moderate or severe incontinence is more likely to require medical treatment than mild incontinence. The VTI parameter 49 showed a significant difference between the two groups (VTI parameter 49 in patients with mild SUI according to MESA SUI score vs. VTI parameter 49 in patients with moderate & severe SUI according to MESA SUI score, mean ± SD 14.06 ± 5.16 vs. 7.54 ± 7.46, *P* = 0.04) (Table 4). VTI parameter 49 describes the displacement of the maximum pressure peak in the anterior compartment, which can be interpreted as the mobility of anterior structures at reflex muscle contraction.

**Table 1:** Demographics and clinical characteristics

	Study Group (N = 31)
Age (years, mean ± SD)	55 ± 11
Gravida (median, range)	3 (1–5)
Parity (median, range)	2 (1–3)
BMI (kg/m^2^, mean ± SD)	27.6 ± 5.8
History of vaginal delivery (n, %)	28 (90)
Postmenopausal (n, %)	18 (58)
PGI-S (mean ± SD)	3.0 ± 0.8
The severity of SUI according to PGI-S	
Mild (n, %)	8 (26)
Severe (n, %)	23 (74)
MESA SUI index (%, mean ± SD)	54 ± 20
MESA UUI index (%, mean ± SD)	15 ± 13
MESA SUI score (mean ± SD)	14.5 ± 5.3
The severity of SUI, according to MESA	
Mild (n, %)	7 (23)
Moderate (n, %)	15 (48)
Severe (n, %)	9 (29)
MESA UUI score (mean ± SD)	2.6 ± 2.4

BMI: Body mass index, PGI-S: Patient Global Impression of Severity and Improvement question, Stress urinary incontinence severity category according to PGI-S scores 1 to 2 is assigned as mild, and 3 to 4 as severe. SUI: stress urinary incontinence, UUI: urge urinary incontinence, MESA: Medical, epidemiologic, and social aspects of aging urinary incontinence questionnaire, Stress urinary incontinence severity category according to MESA: scores of 1 to 9 is assigned as mild, 10 to 18 as moderate, and 19 to 27 as severe

**Table 2:** Examination of bivariate associations between selected VTI parameters and MESA SUI index, MESA SUI score

VTI parameter				MESA SUI Index	MESA SUI Score
No.	Description	Interpretation			
4	The maximum value of posterior gradient (change of pressure per posterior wall displacement in the orthogonal direction to the vaginal channel)	The maximum value of tissue elasticity in the posterior compartment behind the vaginal at a specified location	r	0.373	0.376
			P	0.039	0.037
27	Displacement of the maximum pressure peak in the anterior compartment	Mobility of anterior structures Valsalva maneuver	r	0.366	0.363
			P	0.043	0.044

Simple correlation analyses were used, and Pearson Correlation Coefficient (r) was calculated. Pearson correlation coefficient ‘r’ corresponds to the direction of the relationship. Probability values (P) less than 0.05 were considered significantly different. Only those parameters are shown for which a significant correlation could be found (*P* < 0,05)

**Table 3:** Examine the difference of selected VTI parameters between PGI-S subgroups of SUI patients

VTI parameter				mild SUI according to the PGI-S score (mean ± SD)	severe SUI according to the PGI-S score (mean ± SD)	*P*
No.	Description	Interpretation	Units			
16	The maximum gradient at the perineal body (posterior)	Strength of Level III support (tissue deformation up to 25 mm)	kPa/mm	0.94 ± 1.30	0.22 ± 0.14	0.02
22	Pressure response from a selected location (irregularity 1) at the left side	Hard tissue on the left vaginal wall	kPa	4.96 ± 1.44	3.51 ± 1.55	0.03
23	Pressure response from a selected location (irregularity 2) at the left side	Hard tissue on the left vaginal wall	kPa	5.3 ± 2.44	3.23 ± 1.35	0.01
24	Pressure response from a selected location (irregularity 3) on the right side	Hard tissue on the right vaginal wall	kPa	6.24 ± 1.59	3.97 ± 1.85	0.01
38	Maximum pressure change on the right side at voluntary muscle contraction	Contraction strength of specific pelvic muscle	kPa	7.14 ± 4.64	3.25 ± 3.11	0.02
39	Maximum pressure value on the right side at voluntary muscle contraction	Specified pelvic muscle contractive capability and integrity	kPa	12.48 ± 6.21	6.33 ± 4.44	0.01

To examine the difference between the subgroups of selected VTI parameters in mild/severe incontinence complaints according to the PGI-S question. To assess incontinence severity, the total score of PGI-S is divided into two degrees: scores 1 to 2 are assigned as mild, and 3 to 4 as severe. Only that parameter is shown for which a significant difference could be found (*P* < 0,05)

**Table 4:** Examine the difference of selected VTI parameters between MESA subgroups of SUI patients

VTI parameter				mild SUI according to the MESA SUI score (mean ± SD)	moderate & severe SUI according to the MESA SUI score (mean ± SD)	*P*
**No.**	**Description**	**Interpretation**	**Units**			
49	Displacement of the maximum pressure peak in the anterior compartment	Mobility of anterior structures at reflex muscle contraction	mm	14.06 ± 5.16	7.54 ± 7.46	0.04

To examine the difference between the subgroups of selected VTI parameters in mild vs. moderate & severe SUI. To assess the severity of incontinence, the total score of stress (27) are divided into third degrees: scores 1 to 9 are assigned as mild, 10 to 18 moderate, and 19 to 27 as severe. Only that parameter is shown for which a significant difference could be found (*P* < 0,05)

## Discussion

To our knowledge, this is the first study to describe a correlation between the female pelvic floor biomechanical parameters and the severity of stress urinary incontinence. Our cross-sectional study found significant differences between SUI severity subgroups divided by PGI-S and MESA questionnaire and selected female pelvic floor biomechanical parameters measured by VTI.

SUI is the most common type of UI. The cause of SUI is more intricate than simplistic theories suggesting single anatomical or neurological injuries during childbirth. These injuries expose susceptibility to stress incontinence, which is influenced both genetically (tissue strength, mechanical, and anatomical relationships) and behaviorally (nutrition, smoking, and exercise) [19]. While the Hammock hypothesis currently explains how observed anatomy relates to vaginal, urethral, and bladder function, the Integral Theory prompts us to consider how anatomy may also contribute to bladder overactivity [20–22]. Experts agree that stress urinary incontinence (SUI) can generally be attributed to two primary mechanisms: intrinsic sphincter deficiency and urethral hypermobility.

When assessing a woman with SUI, one of the first steps is to ask the patient to fill out validated questionnaires [23]. The MESA questionnaire is a reliable and validated tool developed as part of the MESA project, an observational study funded by the National Institutes on Aging (NIA) in 1983. Its primary purpose is to identify the urgency- or stress-predominant component of MUI and assess the severity of symptoms [8]. The PGI-S questionnaire asks the patient about the severity of a condition using a 5-point scale, which is not specific to incontinence. However, the severity of the disease can be assessed using the scale: in mild cases, the patient gives a score of 1 or 2, and in severe cases, a score of 3 or 4. In the literature, some articles on studies have used both questionnaires and testing procedures that measure some biomechanical properties of the vagina or pelvic floor. Mariott et al. assessed vaginal pressure profiles before and after prolapse surgery using an intravaginal pressure sensor [24], Pires et al. evaluated maximum voluntary contractions with a perineometer among female athletes [25]. However, a solid-state circumferential catheter or even the Oxford scale can assess simpler biomechanical parameters [26]. Nonetheless, neither of these methods can provide deep enough insight into the pelvic floor’s biomechanical components, which is essential when investigating minor variations or a small number of cases. As shown in the methods section, VTI can measure the appropriate and sufficient biomechanical parameters, as previously demonstrated in the case of POP [12].

Our study demonstrates a positive correlation between the MESA SUI Score and the VTI parameter 4, which refers to the tissues/structures in the anterior compartment at 10–15 mm depth, and parameter 27, corresponding to the displacement of the maximum pressure peak in the anterior compartment. These two parameters align well with urethral hypermobility, a prevalent mechanism in the pathophysiology of SUI [7]. Our results suggest that parameters measured during VTI testing may bring us closer to understanding pathophysiology in a personalized way.

As we aimed to investigate the correlation between the female pelvic floor biomechanical parameters and the severity of stress urinary incontinence, we examined this question using the PGI-S and MESA questionnaires. The PGI-S cannot distinguish between types of incontinence, so all types were investigated in the correlations. Accordingly, several biomechanical parameters of the pelvic floor showed a significant difference when severity was subdivided according to the PGI-S, which we interpreted not as an argument against the use of the PGI-S severity-versus-severity subdivision but as a reason for the different pathophysiological background of the different types of incontinence. In contrast, when the MESA questionnaire was used to divide the study population according to the severity of SUI, only the VTI parameter 49 showed a significant difference between the groups. This parameter can explain the mobility of anterior structures at reflex muscle contraction, which we interpret as further evidence of urethra hypermobility as a pathophysiological factor.

We were unsurprised that the anterior vaginal compartment biomechanical parameters correlate with SUI. The close proximity of the urethra to the anterior vaginal wall allows vaginal assessment by VTI, and also that is it is well-known that hypermobility of the urethra contributes to the pathophysiology of SUI [7]. In contrast, we did not expect to find a correlation between the biomechanical parameters of the posterior vaginal wall and SUI severity. One possible explanation is that the overall weakness of the pelvic floor (both the anterior-posterior parameters are abnormal) may indicate a generally weaker pelvic floor function that may translate into worse SUI. The pathophysiology of SUI is not entirely understood, and our results suggest that VTI may aid in evaluating women with SUI. In addition, VTI technology may help to identify pelvic floor biomechanical weaknesses not identifiable by other methodologies or by urodynamic testing. VTI could further improve the ability to provide appropriately personalized care for SUI patients, thus increasing the chances of a cure for this common disease.

One of our study’s strengths was that we were the first to investigate the association between the female pelvic floor biomechanical parameters and the severity of stress urinary incontinence. We used a non-invasive, easy-to-perform method to assess many critical biomechanical properties of the female pelvic floor. Compared to previous methods, such as manometry or Vaginal Pressure Profile [27], the new method used in the study provides a much more detailed insight into the functioning of the pelvic floor due to the use of several sensors. We have used validated questionnaires to assess UI. Our study has several weaknesses, which need to be offset and improved in further studies. The number of participants was relatively low, resulting in inadequate power to find more minor differences in the biomechanical parameters. In addition, the diagnosis of UI was only established by the questionnaires and not by urodynamics testing. Also, the severity of the SUI was not assessed by pad-test, which is frequently used in research studies but less frequently utilized in everyday clinical practice. A large-scale multicenter study would be required to confirm our results.

## Conclusions

In conclusion, we have shown that female pelvic floor biomechanical parameters, measured by VTI, correlate with the severity of stress urinary incontinence. Considering the relatively small sample size as a limitation of this study, our preliminary data need to be confirmed by more extensive clinical trials. We believe VTI could become a new, easy-to-use method for evaluating women with SUI.

### Electronic supplementary material

Below is the link to the electronic supplementary material.

Supplementary Material 1

## Data Availability

The datasets used and/or analyzed during the current study are available from the corresponding author upon reasonable request.

## List of abbreviations

UI: urinary incontinence
SUI: stress urinary incontinence
UUI: urge urinary incontinence
ISD: intrinsic sphincter deficiency
MESA: Medical, Epidemiologic, and Social Aspects of Aging
PGI-S: Patient Global Impression of Severity question
VTI: Vaginal Tactile Imaging
BI Score: Biomechanical Integrity Score
POP: pelvic organ prolapse
UDI: urodynamic investigation
POP-Q: pelvic organ prolapse quantification system
MUI: mixed urinary incontinence
MID: minimal clinically important difference
BMI: body mass index
NIA: National Institutes on Aging
