# Supplementary material for: Correlation between the female pelvic floor biomechanical parameters and the severity of stress urinary incontinence
Source: BMC Urol. 2023 Nov 30;23:198. doi: 10.1186/s12894-023-01375-7 (PMC10687905; doi:10.1186/s12894-023-01375-7)
Supplement: Supplementary file 1 — Supplementary Material 1 [file 12894_2023_1375_MOESM1_ESM.docx]

VTI parameters

| **No.** | **VTI Test** | **Units** | **Parameter Description** | **Parameter Interpretation** | **Parameter Class** | **Targeting/Contributing**  **Pelvic Structures** | **The mean value of the study population** | **SD** |
| --- | --- | --- | --- | --- | --- | --- | --- | --- |
| 1 | 1 | N | The maximum value of force measured during the VTI probe insertion | Maximum resistance of anterior vs. posterior widening; tissue elasticity at a specified location (capability to resist applied deformation) | Maximum vaginal tissue elasticity at a specified location | Tissues behind the anterior and posterior vaginal walls at 3-15 mm depth | 0.48 | 0.33 |
| 2 | 1 | mJ | Work completed during the probe insertion (Work = Force × Displacement) | Integral resistance of vaginal tissue (anterior and posterior) along the probe insertion | Average vaginal tissue elasticity | Tissues behind the anterior and posterior vaginal walls at 3-15 mm depth | 19.30 | 11.33 |
| 3 | 1 | kPa/mm | The maximum value of anterior gradient (change of pressure per anterior wall displacement in the orthogonal direction to the vaginal channel) | The maximum value of tissue elasticity in the anterior compartment behind the vaginal at a specified location | The maximum value of anterior tissue elasticity | Tissues/structures in the anterior compartment at 10-15 mm depth | 0.67 | 0.55 |
| 4 | 1 | kPa/mm | The maximum value of posterior gradient (change of pressure per posterior wall displacement in the orthogonal direction to the vaginal channel) | The maximum value of tissue elasticity in the posterior compartment behind the vaginal at a specified location | The maximum value of posterior tissue elasticity | Tissues/structures in the anterior compartment at 10-15 mm depth | 0.50 | 0.39 |
| 5 | 1 | kPa | The maximum value of pressure per anterior wall along the vagina | Maximum resistance of anterior tissue to vaginal wall deformation | Anterior tissue elasticity | Tissues/structures in the anterior compartment | 11.30 | 9.14 |
| 6 | 1 | kPa | The maximum value of pressure per posterior wall along the vagina | Maximum resistance of posterior tissue to vaginal wall deformation | Posterior tissue elasticity | Tissues/structures in the posterior compartment | 8.32 | 4.81 |
| 7 | 2 | kPa | Maximum pressure at the area of the pubic bone (anterior) | The proximity of pubic bone to the vaginal wall and perineal body strength | Anatomic aspects and tissue elasticity | Tissues between the vagina and pubic bone; perineal body | 6.45 | 5.70 |
| 8 | 2 | kPa | Maximum pressure at the area of the urethra (anterior) | Elasticity/mobility of the urethra | Anatomic aspects and tissue elasticity | Urethra and surrounding tissues | 4.85 | 2.87 |
| 9 | 2 | kPa | Maximum pressure at the cervix area (anterior) | Mobility of the uterus and conditions of uterosacral and cardinal ligaments | Pelvic floor support | Uterosacral and cardinal ligaments | 4.64 | 3.21 |
| 10 | 2 | kPa | Maximum pressure at the perineal body (posterior) | Pressure feedback of Level III support | Pelvic floor support | Puboperineal, puborectal muscles | 4.56 | 2.95 |
| 11 | 2 | kPa | Maximum pressure at the middle third of the vagina (posterior) | Pressure feedback of Level II support | Pelvic floor support | Pubovaginal, puboanal muscles | 5.75 | 3.05 |
| 12 | 2 | kPa | Maximum pressure at the upper third of the vagina (posterior) | Pressure feedback of Level I support | Pelvic floor support | Iliococcygeal muscle, levator plate | 4.56 | 2.83 |
| 13 | 2 | kPa/mm | The maximum gradient at the area of the pubic bone (anterior) | Vaginal elasticity at the pubic bone area | Anterior tissue elasticity | Tissues between the vagina and pubic bone; perineal body | 0.46 | 0.86 |
| 14 | 2 | kPa/mm | The maximum gradient at the area of the urethra (anterior) | Mobility and elasticity of urethra | Urethral tissue elasticity | Urethra and surrounding tissues | 0.24 | 0.31 |
| 15 | 2 | kPa/mm | The maximum gradient at the cervix area (anterior) | Conditions of uterosacral and cardinal ligaments | Pelvic floor support | Uterosacral and cardinal ligaments | 0.19 | 0.16 |
| 16 | 2 | kPa/mm | The maximum gradient at the perineal body (posterior) | Strength of Level III support (tissue deformation up to 25 mm) | Pelvic floor support | Puboperineal, puborectal muscles | 0.41 | 0.74 |
| 17 | 2 | kPa/mm | The maximum gradient at the middle third of the vagina (posterior) | Strength of Level II support (tissue deformation up to 35 mm) | Pelvic floor support | Pubovaginal, puboanal muscles | 0.27 | 0.34 |
| 18 | 2 | kPa/mm | The maximum gradient at the upper third of the vagina (posterior) | Strength of Level I support (tissue deformation up to 45 mm) | Pelvic floor support | Iliococcygeal muscle, levator plate | 0.22 | 0.17 |
| 19 | 3 | kPa | Maximum pressure at vaginal walls deformation by 7 mm | Hard tissue or tight vagina | Vaginal tissue elasticity | Tissues behind the vaginal walls at 5-7 mm depth | 9.59 | 6.44 |
| 20 | 3 | N | The force applied by anterior and posterior compartments to the probe | Integral strength of anterior and posterior compartments | Vaginal tightening | Tissues behind anterior/ posterior vaginal walls. | 1.73 | 0.70 |
| 21 | 3 | N | The force applied by the entire left and right sides of the vagina to the probe | Integral strength of the left and right sides of the vagina | Vaginal tightening | Vaginal right/left walls and tissues behind them. | 0.95 | 0.46 |
| 22 | 3 | kPa | Pressure response from a selected location (irregularity 1) at the left side | Hard tissue on the left vaginal wall | Irregularity on the vaginal wall | Tissue/muscle behind the vaginal walls on the left side. | 3.88 | 1.65 |
| 23 | 3 | kPa | Pressure response from a selected location (irregularity 2) at the left side | Hard tissue on the left vaginal wall | Irregularity on the vaginal wall | Tissue/muscle behind the vaginal walls on the left side. | 3.76 | 1.92 |
| 24 | 3 | kPa | Pressure response from a selected location (irregularity 3) on the right side | Hard tissue on the right vaginal wall | Irregularity on the vaginal wall | Tissue/muscle behind the vaginal walls on the right side. | 4.55 | 2.04 |
| 25 | 4 | N | Integral force change in the anterior compartment at Valsalva maneuver | Pelvic function at Valsalva maneuver | Pelvic function | Multiple pelvic muscles | 1.10 | 0.67 |
| 26 | 4 | kPa | Maximum pressure change in the anterior compartment at Valsalva maneuver. | Pelvic function at Valsalva maneuver | Pelvic function | Multiple pelvic muscles | 7.03 | 6.32 |
| 27 | 4 | mm | Displacement of the maximum pressure peak in the anterior compartment | Mobility of anterior structures Valsalva maneuver | Pelvic function | Urethra, pubovaginal muscle; ligaments | 1.75 | 5.95 |
| 28 | 4 | N | Integral force change in the posterior compartment at Valsalva maneuver | Pelvic function at Valsalva maneuver | Pelvic function | Multiple pelvic muscles | 1.09 | 0.65 |
| 29 | 4 | kPa | Maximum pressure change in the posterior compartment at Valsalva maneuver. | Pelvic function at Valsalva maneuver | Pelvic function | Multiple pelvic muscles | 5.34 | 3.05 |
| 30 | 4 | mm | Displacement of the maximum pressure peak in the posterior compartment | Mobility of posterior structures Valsalva maneuver | Pelvic function | Anorectal, puborectal, and pubovaginal muscles; ligaments | 0.52 | 4.09 |
| 31 | 5 | N | Integral force change in the anterior compartment at voluntary muscle contraction | Integral contraction strength of pelvic muscles along the vagina | Pelvic function | Puboperineal, puborectal, pubovaginal, and ilicoccygeal muscles; urethra | 1.09 | 0.70 |
| 32 | 5 | kPa | Maximum pressure change in the anterior compartment at voluntary muscle contraction | Contraction strength of specified pelvic muscles | Pelvic function | Puboperineal, puborectal and pubovaginal muscles | 14.25 | 13.92 |
| 33 | 5 | kPa | Maximum pressure value in the anterior compartment at voluntary muscle contraction. | Static and dynamic peak support of the pelvic floor | Pelvic function | Puboperineal and puborectal muscles | 19.15 | 14.99 |
| 34 | 5 | N | Integral force change in the posterior compartment at voluntary muscle contraction | Integral contraction strength of pelvic muscles along the vagina | Pelvic function | Puboperineal, puborectal, pubovaginal, and ilicoccygeal muscles | 1.19 | 0.64 |
| 35 | 5 | kPa | Maximum pressure change in the posterior compartment at voluntary muscle contraction | Contraction strength of pelvic muscles at a specified location | Pelvic function | Puboperineal, puborectal and pubovaginal muscles | 8.73 | 5.71 |
| 36 | 5 | kPa | Maximum pressure value in the posterior compartment at voluntary muscle contraction. | Static and dynamic peak support of the pelvic floor | Pelvic function | Puboperineal and puborectal muscles | 13.19 | 6.76 |
| 37 | 6 | N | Integral force change in the right side at voluntary muscle contraction | Integral contraction strength of pelvic muscles along the vagina | Pelvic function | Puboperineal, puborectal, and pubovaginal muscles | 0.56 | 0.43 |
| 38 | 6 | kPa | Maximum pressure change on the right side at voluntary muscle contraction | Contraction strength of specific pelvic muscle | Pelvic function | Puboperineal or puborectal or pubovaginal muscles | 4.25 | 3.95 |
| 39 | 6 | kPa | Maximum pressure value on the right side at voluntary muscle contraction | Specified pelvic muscle contractive capability and integrity | Pelvic function | Puboperineal or puborectal muscles | 7.91 | 5.64 |
| 40 | 6 | N | Integral force change in the left side at voluntary muscle contraction | Integral contraction strength of pelvic muscles along the vagina | Pelvic function | Puboperineal, puborectal, and pubovaginal muscles | 0.52 | 0.41 |
| 41 | 6 | kPa | Maximum pressure change on the left side at voluntary muscle contraction | Contraction strength of specific pelvic muscle | Pelvic function | Puboperineal or puborectal or pubovaginal muscles | 4.20 | 4.04 |
| 42 | 6 | kPa | Maximum pressure value on the left side at voluntary muscle contraction | Specified pelvic muscle contractive capability and integrity | Pelvic function | Puboperineal or puborectal muscles | 7.61 | 5.90 |
| 43 | 7 | kPa/s | Anterior absolute pressure change per second for maximum pressure at involuntary relaxation | Innervation status of specified pelvic muscles | Innervations status | Levator ani muscles | -1.75 | 2.02 |
| 44 | 7 | %/s | Anterior relative pressure change per second for maximum pressure at involuntary relaxation | Innervation status of specified pelvic muscles | Innervations status | Levator ani muscles | -8.75 | 6.71 |
| 45 | 7 | kPa/s | Posterior absolute pressure change per second for maximum pressure at involuntary relaxation | Innervation status of specified pelvic muscles | Innervations status | Levator ani muscles | -1.10 | 1.03 |
| 46 | 7 | %/s | Posterior relative pressure change per second for maximum pressure at involuntary relaxation | Innervation status of specified pelvic muscles | Innervations status | Levator ani muscles | -8.98 | 6.46 |
| 47 | 8 | N | Integral force change in the anterior compartment at reflex pelvic muscle contraction (cough) | Integral pelvic function at reflex muscle contraction | Pelvic function | Multiple pelvic muscles | 1.67 | 0.80 |
| 48 | 8 | kPa | Maximum pressure change in the anterior compartment at reflex pelvic muscle contraction (cough). | Contraction strength of specified pelvic muscles | Pelvic function | Multiple pelvic muscles | 9.09 | 4.05 |
| 49 | 8 | mm | Displacement of the maximum pressure peak in the anterior compartment | Mobility of anterior structures at reflex muscle contraction | Pelvic function | Urethra, pubovaginal muscle; ligaments | 9.01 | 7.52 |
| 50 | 8 | N | Integral force change in the posterior compartment at reflex pelvic muscle contraction (cough) | Integral pelvic function at reflex muscle contraction | Pelvic function | Multiple pelvic muscles | 1.77 | 0.90 |
| 51 | 8 | kPa | Maximum pressure change in the posterior compartment at reflex pelvic muscle contraction (cough). | Contraction strength of specified pelvic muscles | Pelvic function | Multiple pelvic muscles | 8.72 | 4.05 |
| 52 | 8 | mm | Displacement of the maximum pressure peak in the posterior compartment | Mobility of anterior structures at reflex muscle contraction | Pelvic function | Anorectal, puborectal, and pubovaginal muscles; ligaments | 6.76 | 6.11 |
